# The protective effects of chronic intermittent hypobaric hypoxia pretreatment against collagen-induced arthritis in rats

**DOI:** 10.1186/s12950-015-0068-1

**Published:** 2015-03-25

**Authors:** Min Shi, Fang Cui, Ai-Jing Liu, Hui-Jie Ma, Ming Cheng, Shu-Xia Song, Fang Yuan, De-Pei Li, Yi Zhang

**Affiliations:** Department of Physiology, Hebei Medical University, Shijiazhuang, 050017 China; Department of Clinical Laboratory, The Second Hospital of Hebei Medical University, Shijiazhuang, 050017 China; Department of Immune and Rheumatism, The Second Hospital of Hebei Medical University, Shijiazhuang, 050017 China; Department of Immunology, Hebei Medical University, Shijiazhuang, 050017 China; Department of Critical Care, The University of Texas, MD Anderson Cancer Center, Houston, TX 77030 USA; Hebei Key Laboratory of Medical Biotechnology, Shijiazhuang, 050017 China; Hebei Collaborative Innovation Center for Cardio-Cerebrovascular Disease, Shijiazhuang, Hebei 050000 PR China

**Keywords:** Chronic intermittent hypobaric hypoxia, Rheumatoid arthritis, T lymphocytes, Th1/Th2, HIF-1α, NF-κB, Rat

## Abstract

**Objective:**

To explore the immunological mechanisms underlying the effect of chronic intermittent hypobaric hypoxia (CIHH) pretreatment on collagen-induced arthritis (CIA) in rat.

**Methods:**

Fifty-four adult male Sprague–Dawley rats were used in the experiment. Arthritis in CIA rats (n=18) was induced by injection of collagen. The CIHH+CIA rats (n=18) were treated with CIHH (simulated 3000 m altitude, 5 hours per day for 28 days, PO_2_=108.8 mmHg) before CIA. The control rats (n=18) were not given any treatment.

**Results:**

(1) Incidence rate of CIA in CIHH+CIA rats was significantly lower than that in CIA rats (P<0.05). (2) The paw thickness and arthritis index (AI) value in CIHH+CIA rats were lower than those in CIA rats (P<0.05). (3) The hyperplasia with inflammatory infiltration in synovial tissue of joints in CIHH+CIA rats was much alleviative compared with CIA rats. (4) TNF-α, IFN-γ, IL-4 and IL-17 in synovial tissue of joint and serum in CIHH+CIA rats were decreased compared with CIA rats (P<0.05). (5) The number of CD4-positive T-lymphocytes and the ratio of CD4/CD8 T-lymphocytes in peripheral blood in CIHH+CIA rats were lower than those in CIA rats (P<0.05). (6) The protein expression of HIF-1α and NF-κB in synovial tissue of joint in CIHH+CIA rats was decreased compared with CIA rats (P<0.05).

**Conclusion:**

CIHH pretreatment has a protective effect against collagen-induced arthritis in rat through down-regulation of HIF-1α and NF-κB, inhibition of inflammatory cytokines TNF-α and IL-17, and balance in CD4/CD8 and Th1/Th2 T lymphocytes.

Rheumatoid arthritis (RA), an immunological abnormality induced inflammatory disease, is characterized as chronic, symmetrical, multi-synovium arthritis and pathological changes outside joints. The collagen-induced arthritis (CIA) rat model is a good experimental model for study mechanisms of arthritis and is demonstrated to have cellular immunity abnormality in arthritis [1]. Because the pathological changes in the blood and the articular tissue in the model are similar to those in human RA, the CIA rat model is widely used in studying human arthritis. Up to date, the mechanism of RA is not fully understood and there is no specific and effective therapy for RA treatment.

Accumulating evidence showed that chronic intermittent hypobaric hypoxia (CIHH) has beneficial effects on the body. For example, CIHH protects heart, central nervous system, and liver against ischemia/reperfusion or hypoxia/reoxygenation injuries [2-4]. Also, CIHH exerts anti-hypertension effect [5]. Furthermore, CIHH has been widely used in sports training to enhance the tolerance of organs and tissues to anoxia [6]. It was reported that CIHH had a positive treatment effect on bronchial asthma, which suggests that CIHH has a beneficial action on immune function [7]. Our previous study has shown that CIHH treatment recovered the immunological dysfunction induced by acute hypoxia through protecting lymphocytes in thymus and spleen and balancing the disturbance of T-lymphocytes subgroups [8]. Recently, our group found that CIHH pre-treatment could diminish the incidence of CIA and alleviate the symptoms of CIA in rats, which may related to CIHH enhancing apoptosis in synovial cells and T lymphocytes [9]. The aforementioned suggest that CIHH has beneficial effect on immune system by modulating the immunological function and lymphocytes activity. But the detail immunological mechanism underlying protection of CIHH against CIA remains unclear. So we proposed a hypothesis that CIHH protects CIA through inhibiting inflammatory reaction and keeping the balance of lymphocytes activity. The primary objective of this study was to investigate the protective effect of CIHH against CIA and the anti-inflammation action of lymphocytes in the protective effect of CIHH, and the second objective was to confirm the signaling molecule like HIF-1α and NF-κB in CIHH effect. The present study demonstrated that CIHH pretreatment has a protective effect against collagen-induced arthritis in rat through down-regulation of HIF-1α and NF-κB, inhibition of inflammatory cytokines TNF-α and IL-17, and balance in CD4/CD8 and Th1/Th2 T lymphocytes.

## Materials and methods

### Reagents

Bacillus Calmette-Guerin Vaccine (BCG) frozen powder (80 mg/vial) was purchased from Beijing institute of biological products. Complete Freund’s adjuvant (10 ml/vial) and Bovine collagen type II (C-7806) were purchased from Sigma Co. USA. Tumor necrosis factor-α (TNF-α), IFN-γ, IL-4 and IL-17 ELISA kits were purchased from Shanghai Sengxiong Technological Co. China. Fluorescein isothiocyanate (FITC)-conjugated anti-rat CD4 antibody and allophycocyanin (APC)-conjugated anti-rat CD8 antibody were purchased from eBioscience USA. Anti-rat TNF-α, IFN-γ, IL-4 and IL-17, monoclonal anti-rat HIF-1α and NF-κB, and horseradish peroxidase (HRP) were purchased from Santa Cruz Biotechnology, USA. SP9001 kit and DAB (3,3’-diaminobenzidine) color reagent were purchased from Beijing Zhongshan Jinqiao Biotech Co. Ltd. China. BCA kit was purchased from Pierce Co. USA.

### Animal group and protocols

All experiments were carried out in compliance with the Guide for the Care and Use of Laboratory Animals (National Research Council, 1996) and was reviewed and approved by the Ethics Committee for the Use of Experimental Animals at Hebei Medical University. Fifty-four adult male Sprague–Dawley rats (provided by Experimental Animal Center of Hebei Medical University, body weight 130-150 g,) were randomly divided into 3 groups: CIA, CIHH plus CIA (CIHH+CIA) and Control groups. CIA rats (n=18) received collagen injection to induce CIA. CIHH+CIA rats (n=18) received CIHH treatment (simulating 3000 m altitude, 5 hours per day for 28 days, PO_2_=108.8 mmHg) in a hypobaric chamber before collagen injection. Control rats (n=18) received local injection of physiological saline.

The healthy condition and physical activity of the rats were monitored daily, and the body weight and paw thickness were measured and recorded every week. The blood samples from carotid artery and synovial tissue samples in the joints of hind paws of anesthesia rats were collected at end of experiments. The rats were sacrificed with over-dose of pentobarbital sodium (100 mg/kg, i.v) after samples collection. In our study, preparation of CIA rats, collection of sample and measurement of outcomes were performed by a same skillful person respectively to reduce subjective and artificial errors.

### Protocols

The whole duration for animal treatment lasts 49 days. For CIHH+CIA rats, CIHH treatment was given from 1st day to 28th day, a collagen injection was given at 29th day, and the samples of blood and synovial tissue in joints of hind paws were collected to assess the outcomes at 49th day. For CIA rats, a collagen injection was given at 29th day, and the samples of blood and synovial tissue were collected at 49th day. For Control rats, a physiological saline injection was given at 29th day, and the samples of blood and synovial tissue were collected at 49th day.

### Induction of arthritis

Bovine collagen type II (1 mg/ml) was dissolved in 0.1 M acetic acid, and kept overnight at 4°C, then emulsified fully with BCG (10 mg/ml) and equivalent volume of complete Freunds adjuvant (CFA). The emulsified agent containing collagen of 0.2 ml was injected in the back or at the base of the tail, with 6–8 points per rat, to induce arthritis. For control rats, equivalent volume of physiological saline solution for collagen preparation was injected in multiple points.

Arthritic Index (AI) was determined by the degree of redness and swelling of the joints and was used to evaluate the severity of arthritis [10]. Grade 0 of AI represents no edema and swelling in the joint; Grade 1 represents slight edema and erythema limited to the foot and/or ankle; Grade 2 represents edema and erythema toes and most joints of the ankles; Grade 3 represents severe edema and erythema paw below ankle joint; Grade 4 represents edema and erythema of all paws including ankle joint. The model of arthritis was considered successful when AI > 5. The evaluation was performed each week.

### CIHH treatments

In the CIHH treatment, a vacuum pump was used to pump air out of the hypobaric chamber, resulting in the decrease of chamber pressure below the atmosphere. At same time, fresh air flow into the chamber through a small ventilation hole to keep enough fresh air for the animal breath. For the safety of animal, the decompression speed was controlled below 2.5 m/s by a ventilation valve. It is about 20 min for the air pressure in the chamber to reach 108.8 mmHg that represents the air pressure at 3000 m altitude. After 5 hours hypobaric hypoxia simulating 3000 m altitude, the pump is turned out and the chamber pressure was increased gradually to the atmosphere pressure (The increase speed is below 2.5 m/s).

### Preparation of articular synovium

The rats were anesthetized with pentobarbital sodium (50 mg/kg ip) and fixed in supine position. The skin on the legs was cut longitudinally to expose the area of knee joint. The ligament and soft tissues above the patella were cut off. Blunt dissection was performed to separate synovial and fibrous layers and to fully expose synovial tissues. One of synovial tissue was cut off and put into 4% paraformaldehyde for histopathology examination and immunohistochemical staining. Another synovial tissue was quickly put into liquid nitrogen and kept for 15 min, and then shift into −80°C for measuring the protein expression of HIF-1α and NF-κB by using Western blots method.

### Histopathology of articular synovium

After routine dehydration and paraffin embedding, the synovium tissue was cut to 5 μm-thick sections that were used to haemotoxylin-eosin (HE) staining and immunohistochemical staining. Pathological hyperplasia, inflammatory infiltration, and pannus development in articular synovium were observed under optical microscope after HE staining.

### Immunohistochemical staining and Immunohistochemisty score in articular synovium

Immunohistochemical staining of streptavidin-perosidase for TNF-α, IFN-γ, IL-4 and IL-17 were performed according to the procedures described by the manufacture of the staining kit. The results were judged based on chromogenic reaction after staining. A brownish yellow color indicated positive staining in the cytoplasm of the synovial tissue. At least five visual fields was observed in one section and the percentage of the positive was calculated as the brownish yellow colored cells over total number of cells.

We also determined the Immunohistochemisty Score (IHS) as the following [11]: IHS = A×B in which A stands for positive cell count grading (0~1% = grade 0, 1~10% = grade 1, 10~50% = grade 2, 50~80% = grade 3, and 80~100% = grade 4), and B stands for intensity of positive cell coloration (0 represents negative, 1 represents weak positive, 2 represents positive, and 3 represents strong positive).

### Measurement of TNF-α, IFN-γ, IL-4 and IL-17 in serum

Blood sample (3 ml for each rat) was collected from intubation in common carotid artery and centrifuged for 5 min (3000 r/min) to get serum. The serum was kept at −20°C for using. Enzyme linked immunosorbent assay (ELISA) was used to determine the content of TNF-α, IFN-γ, IL-4 and IL-17 in the serum. According to the procedures indicated in kit, optical density value was recorded at 450 nm on the ELISA (Multiskan Spectrum1500, TEC Co., US) 15 min after the cessation of the reaction. The concentration of TNF-α, IFN-γ, IL-4 and IL-17 was obtained from standard log-log graph.

### Determination of subsets of CD4 and CD8 on the T-lymphocyte in the peripheral blood

Blood sample was collected in the same way as mentioned above. Flow cytometry was used to determine the subsets of lymphocyte CD4 and CD8. One ml blood was mixed fully with the anti-coagulant in a ratio of 1:9 the anti-coagulating vacuum tube. Corresponding fluorescence labeling monoclonal antibodies against CD4 (5 μl) and CD8 (5 μl) were added into the tubes containing 100 μl aliquot of blood and anticoagulant mixture, respectively. The contents in the tubes were mixed vertically and kept for 20 min at room temperature in the dark. Then, 50 μl hemolysin was added into each tube. After mixture and the tubes were mixed and kept for 10 min in the dark, pure water 500 μl was added. After hemolysis completed and phosphate buffered saline (PBS, 3 ml) added, the tube was centrifuged for another 3–5 min at 1200 r/min. The supernatant was decanted and the sedimented cells were obtained and kept at 4°C. At least 10,000 cells for each sample were obtained and analyzed by Cellquest software.

### Determination of HIF-1α and NF-κB in articular synovium tissue

Western blot technique was used to determine the expression of HIF-1α and NF-κB proteins in articular synovium tissue. The synovial tissues were homogenated and centrifuged at 12000 r/min for 5 min at 4°C. Supernatant was collected and protein concentration in supernatant was quantified using BCA method. The supernatant, each with equal protein loading, was subjected to SDS-PAGE and electrotransferred to the membrane. Blots were incubated firstly with the primary antibody against NF-κB (Santa Cruz Biotechnology. USA, 1:250 dilution) or HIF-1α (Santa Cruz Biotechnology.USA, 1:200 dilution) for 2 h and then with a secondary antibody against IgG (Santa Cruz Biotechnology.USA, 1:10 000 dilution) for 1 h after washed with PBS-Tween for three times. Blots were developed using DAB color reagent. The protein in blots was quantified by densitometry using Quantity one analysis software and normalized to scanning signals of GADPH.

### Statistics

The data were expressed as mean ± SEM. To compare the differences of body weight, paw thickness, AI, IHS, CD4 and CD8-positive T-lymphocytes in peripheral blood, and Inflammatory cytokines such as TNF-α, IFN-γ, IL-4 and IL-17 in synovium and serum, as well as HIF-1α and NF-κB in synovium tissue within the experimental groups, one-way Analysis of Variances (ANOVA) followed by a Dunnett’s post hoc test was performed. For the enumeration data (the incidence rate of CIA), expressed as percentage, was analyzed by *x*^2^ test. *P* < 0.05 was considered statistically significant.

## Results

### General state of animals

**Figure 1:**
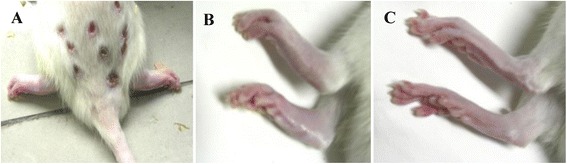
**Rat model of collagen-induced arthritis (CIA). A**. Scab with ulcer on the tail and back in CIA rat; **B**. Red swelling of the paws in CIA rat; **C**. Normal paws in control rat.

The rats in control group were healthy as indicated by increased body weight, lustrous hair, normal physical activity, and free movement, while the rats treated with collagen were unhealthy, as indicated by lower gain of body weight, weariness, withered hair, and reduced physical activities. Small inflammatory ulcers appeared at the back and tail skin surrounding the injection site in the third day after collagen injection. The ulcers experienced hard crusts formation, drop off, and healing during 7 to 10 days after collagen injection (Figure 1A). The skin surrounding the injection sites were red with slight swelling in the fourteenth day after collagen injection. The swelling in the ankles, especially hind ankles, was gradually aggravated and reached to the peak in the Eighteenth day (Figure 1B). Then the swelling was alleviated gradually but the joints became stiff and the movements of the limbs were limited.

**Figure 2:**
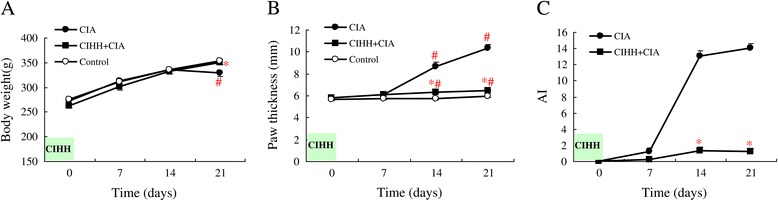
**Chronic intermittent hypobaric hypoxia pretreatment improves body weight, paw thickness and Arthritic Index in collagen-induced arthritis rat.** Chrinic intermittent hypobaric hypoxia (CIHH) pretreatment antagonized the decrease of body weight **(A)** and the increase of paw thickness **(B)**, and decreased the Arthritic Index **(C)** in collagen-induced arthritis rat. AI: arthritis index; CIA: collagen-induced arthritis group; CIHH+CIA: CIHH treated CIA group; Data were expressed as mean ± SEM, *n* = 6 for each group, *p<0.05 *vs* CIA, #*p<*0.05 *vs* control.

The body weight of CIA rats was significant lower in the twenty-first day after collagen injection compared with CIHH+CIA and control rats (*P* < 0.05, Figure 2A). There was no significant difference of body weight between CIHH+CIA and control rats (*P* > 0.05, Figure 2A).

### The effect of CIHH on the incidence rate of CIA

In CIHH+CIA group, 4 out of 20 (20%) rats developed collagen-induced arthritis. However, 14 out of 20 (70%) rats in CIA group developed collagen-induced arthritis. The incidence rate of collagen-induced arthritis in CIHH+CIA group (20%) was significantly lower than that in CIA group (*P* < 0.05, Table 1).

**Table 1:** **Effect of chronic intermittent hypobaric hypoxia (CIHH) pre-treatment on incidence rate in collagen-induced arthritis rat**

**Group**	**Number of cases**	**Number of incidence**	**Incidence rate (%)**
CIA	20	14	70
CIHH+CIA	20	4	20*

x^2^=10.101, *P < 0.05 *vs* CIA group.

### The effect of CIHH on paw thickness and AI in rats

The paw thickness in CIA rats was significantly increased in the fourteenth day after collagen injection (8.66 ± 0.44 mm) compared with that before collagen injection (6.08 ± 0.10 mm, *P* < 0.05). The paw thickness of CIHH+CIA rats was lower than that in CIA rats from the fourteenth day to the twenty-first day after collagen injection (*P* < 0.05, Figure 2B).

AI value in CIA rats was increased significantly in the fourteenth day after collagen injection (*P* < 0.05). AI in CIHH+CIA rats was lower than that in CIA rats from the fourteenth day to the twenty-first day after collagen injection (*P* < 0.05, Figure 2C).

### The effect of CIHH on articular synovium pathology in rats

**Figure 3:**
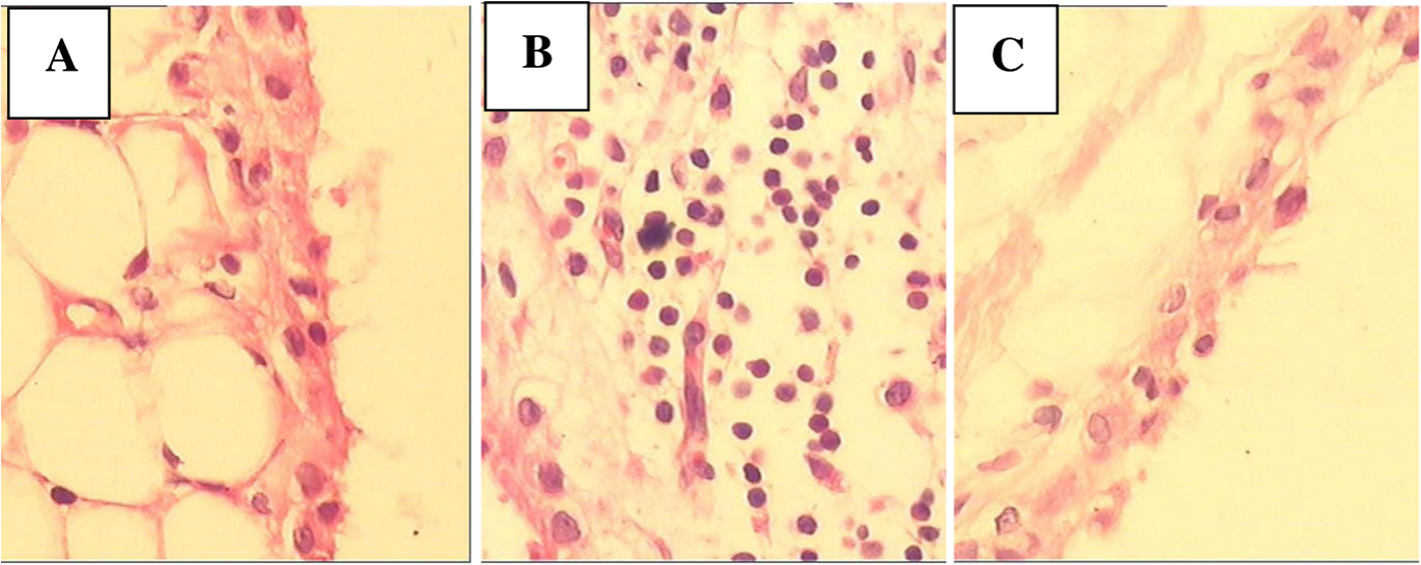
**Effect of chronic intermittent hypobaric hypoxia (CIHH) pretreatment on pathologic morphology of synovial tissue in collagen-induced arthritis rat. A**: Control rats displayed normal synovial tissue; **B**: Collageniinduced arthritis (CIA) rats displayed obvious hyperplasia with inflammatory infiltration in liner and underlayer of liner; **C**: CIHH treated CIA (CIHH+CIA) rats did not displayed significant difference from control rats. (H-E staining × 100).

Normal histology of articular synovium has 1–2 layers of synovial lines, with regular epithelial cells in the flat synovium, and no inflammatory infiltration or angiogenesis (Figure 3A). The articular synovium in CIA rats displayed obvious hyperplasia with inflammatory infiltration in liner and underlayer of liner (Figure 3B). There was no significant difference of synovium architecture between CIHH+CIA and control rats (Figure 3C).

### The effect of CIHH on CD4 and CD8-positive T-lymphocytes in blood of rats

The CD4 -positive T-lymphocytes were increased (30.58 ± 1.59), while the CD8-positive T-lymphocytes decreased remarkably (22.47 ± 1.63) in peripheral blood of CIA rats. The ratio of CD4/CD8 T-lymphocytes in CIA rats was elevated compared with CIHH+CIA rats (*P<*0.05). However, there was no significant difference of CD4 and CD8 T-lymphocytes between control and CIHH+CIA rats (*P* >0.05, Table 2).

**Table 2:** **Effect of chronic intermittent hypobaric hypoxia (CIHH) pre-treatment CD4 and CD8 in blood of collagen-induced arthritis rat**

**Group**	**CD4 (%)**	**CD8 (%)**	**CD4/CD8**
CIA (n=6)	30.58±1.59^#^	22.47±1.63	1.33±0.09^#^
CIHH+CIA (n=6)	24.62±0.83*	25.08±1.23	0.99±0.03*
Control (n=6)	25.42±1.18	26.02±0.89	0.98±0.04

Data were expressed as mean±SEM; *P < 0.05 *vs* CIA; ^#^ *P* < 0.05 *vs* Control.

### The effect of CIHH on expression of TNF-α, IFN-γ, IL-4 and IL-17 in synovial tissue

**Figure 4:**
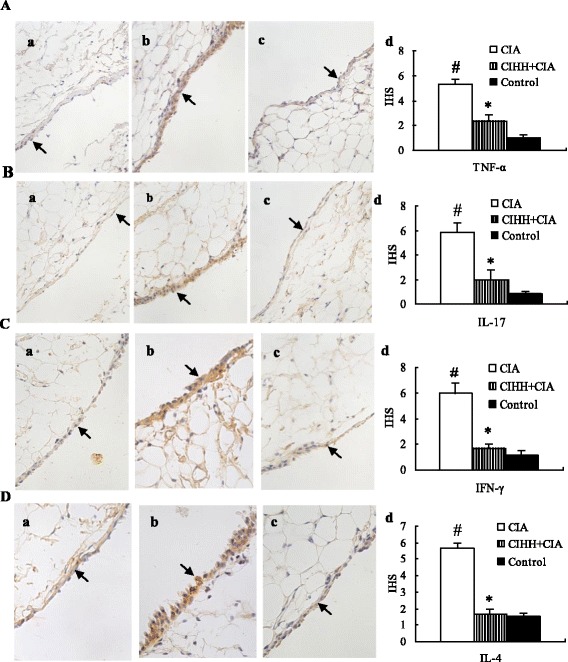
**Effect of chronic intermittent hypobaric hypoxia (CIHH) pretreatment on TNF-α (A), IL-17 (B), IFN-γ (C) and IL-4 (D) protein expression in synovial tissue of collagen-induced arthritis rat.** Representative photomicrographs of protein expression in synovial tissue of Control (a), CIA (b), CIHH+CIA (c) and IHS (d). Brown staining indicates protein expression in cytoplasm (arrow). CIA: collagen-induced arthritis groups; CIHH+CIA: CIHH treated CIA group; HIS:Immunohistochemistry score of protein expression; Data were expressed as mean ± SEM, *n* = 6 for each group, * *p<*0.05 *vs* CIA,# *p<*0.05 *vs* Control (Immunohistochemistry staining × 400).

The expression of TNF-α, IFN-γ, IL-4 and IL-17 in synovial tissue of CIHH+CIA rats was decreased markedly compared with CIA rats (*P* < 0.05). Compared with control rats, however, expression of IL-17 in CIHH+CIA rats was increased (*P* < 0.05, Figure 4).

### The effect of CIHH on serum TNF-α, IFN-γ, IL-4 and IL-17 concentration in rats

The serum TNF-α, IFN-γ, IL-4, the ratio of IFN-γ/IL-4 and IL-17 in CIHH+CIA rats were decreased significantly compared with CIA rats (*P* < 0.05), but were not changed significantly compared with control rats (*P* > 0.05, Table 3).

**Table 3:** **Effect of chronic intermittent hypobaric hypoxia (CIHH) pre-treatment on TNF-α, IL-17, IFN-γ, IL-4 and IFN-γ/ IL-4 in serum of collagen-induced arthritis rat**

**Group**	**TNF-α (pg/ml)**	**IL-17 (pg/ml)**	**IFN-γ (pg/ml)**	**IL-4 (pg/ml)**	**IFN-γ/IL-4**
CIA (n=6)	4.28±0.49^#^	12.95±0.59^#^	9.89±0.31^#^	10.75±0.27	0.92±0.02^#^
CIHH+CIA (n=6)	2.21±0.36^*^	10.85±0.65^*^	7.53±0.27^*^	9.46±0.41^*^	0.80±0.05^*^
Control (n=6)	2.25±0.22	10.54±0.41	7.59±0.56	9.52±0.45	0.81±0.04

Data were expressed as mean±SEM; *P <0.05 *vs* CIA; ^#^ *P* <0.05 *vs* Control.

### The effect of CIHH on protein expression of HIF-1α and NF-κB in synovium tissue

**Figure 5:**
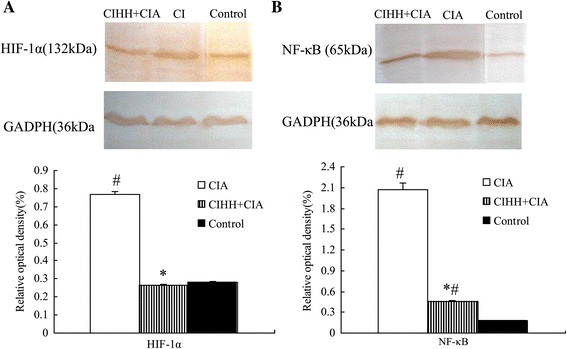
**Chronic intermittent hypobaric hypoxia pretreatment decreases HIF-1α and NF-κB protein expression of synovial tissue in collagen-induced arthritis rat.** Chronic intermittent hypobaric hypoxia (CIHH) pretreatment prevented the protein over expression of HIF-1α **(A)** and NF-κB **(B)** in synovial tissue of collagen-induced arthritis rat. Representative photographs of protein expression in synovial tissue by western blots technique (above); Summaries of densitometric scanning of protein normalized to GADPH (bottom); CIA: collagen-induced arthritis groups; CIHH + CIA: CIHH pretreatment group; Data were expressed as mean ± SEM, n = 6 for each group, * *P* < 0.05 vs CIA,# *P* < 0.05 vs Control.

The protein expression of HIF-1α in synovium tissue in CIHH+CIA rats was significantly lower than that in CIA rats (*P*<0.05), but was not different compared with Control rats (*P>*0.05, Figure 5A).

The protein expression of NF-κB in synovium tissue in CIHH+CIA rats was significantly lower than that in CIA rats (*P*<0.05), but higher than that in Control rats (*P*<0.05, Figure 5B).

## Discussion

This is the first study to investigate the underlying immunological mechanism for the protective effect of CIHH on CIA rats. We found that pre-treatment of CIHH significantly decreased the prevalence of CIA and effectively alleviated the symptoms of CIA, such as reducing AI value and paw thickness and relieving inflammatory infiltration in synovial tissue. Furthermore, CIHH treatment inhibited the increasing of IL-4, TNF-α, IL-17 in serum and synovium tissue, kept the balance of CD4/CD8 T-lymphocytes and IFN-γ/IL-4 in blood, and inhibited the protein expression of HIF-1α and NF-κB in synovium tissue in CIA rats. These data suggest that CIHH treatment exerts beneficial effect on CIA in rats, which was related to inhibition of inflammatory cytokines, regulation of lymphocyte immunity and HIF-1α-NF-κB signaling pathway.

The ratio of Th1/Th2 lymphocytes reflects balance state of cellular immune in the body. It was reported that the inbalance of Th1/Th2 was existed in RA synovial tissue and blood with inflammatory cytokines IFN-γ and TNF-а from Th1 cells predominance [12]. The Th17 cell, differentiated from CD4-positive T cells, belongs to a subgroup of T-lymphocyte and contributes importantly to inflammation and autoimmune diseases [13]. The IL-17, secreted by Th17 cells, is a major effective factor to induce the production and release of many inflammatory cytokines such as chemotactic factors [14]. Previous studies have shown that IL-17 is involved in RA and has a synergetic effect with TNF-α on inflammation enhancement [15]. The IL-17 antagonist had been proved to have a therapeutic effect on CIA animals [16]. The result of present study showed CIHH pretreatment inhibited the increase of IFN-γ, IL-4 and IL-17, regulated the ratio of IFN-γ/IL-4 (represents Th1/Th2 lymphocytes) and ratio of CD4/CD8 T-lymphocytes in CIA rats, which indicates that CIHH protects rat against CIA through keeping the homeostasis of lymphocytes.

It has been known that proinflammatory cytokine-mediated inflammatory reaction is involved in the pathological changes of RA synovium [17]. TNF-α is one of vital proinflammatory cytokines in the RA pathogenesis and is produced by macrophagocyte, CD4-positive T cells, and natural killer cells. It has been reported that TNF-α exerts many effects in the RA pathological process. For example, TNF-α promote the adhesion and permeation of leukocyte with vascular endothelium, which results in local inflammation [18]. Also, TNF-α enhances the bone destruction, absorption, and fibroblast hyperplasia, an effect inhibit the synthesis of bone collagen [19]. Furthermore, TNF-α increases the release of growth factor from synovium, endothelium, and fibroblast to promote pannus formation of TNF-α [20]. The TNF-α level increases markedly in serum and joint fluid of RA patient [21,22]. On the other hand, anti-TNF-α medicine had therapeutic effect on ameliorating both inflammation and joint damage in rheumatoid arthritis patient [23]. Also, IL-12 and IFN-γ from Th1 cells and IL-17 from Th17 cells act as proinflammatory cytokines [24,14]. In the present study, we found that CIHH treatment reduced the increase of TNF-α, IFN-γ and IL-17 in serum and synovial tissue in CIA rats, suggesting CIHH may protect rat against CIA through inhibition of inflammatory reaction.

Nuclear factor-kappa B (NF-κB) and hypoxia inducible factor (HIF-1α), two important transcription factors related to inflammation and hypoxia, have been proved to be involved in RA [25,26]. Both factors play key role on regulation of inflammation gene in RA. NF-κB regulates growth and differentiation of immune cell, participates in regulation of immune-related and inflammation-related genes, and highly expresses in pathologic synovium tissue of RA patient [27]. Hypoxic microenvironment in RA contributes to the characterized angiogenesis, pannus and inflammation process, in which the abnormal activation of HIF-1α pathway was closely related to inflammation development [28]. While inflammation cytokines such as IL-1β and TNF-α can promote the transcription of HIF-1 [29]. Activated HIF-1α promotes angiogenesis and inflammation of joint synovium, resulting in joint cartilage and bone damage [30]. In general, NF-κB and HIF-1 pathways take action independently. On the other hand, a “cross-talk” exists between NF-*κ*B and HIF-1 resulting in interdependence of them under disease states, such as ischaemia, pulmonary hypertension, cancer, and a variety of acute or chronic inflammatory diseases [31,32]. The result of this study showed that pretreatment of CIHH could abate the overexpression of NF-κB and HIF-1 in RA rats, which suggests that CIHH protects rat against RA through modulating signal pathways of NF-κB and HIF-1α.

As stress stimulation, hypoxia is a double-edged sword to the body. In this regard, severe hypoxia is involves in the genesis and prognosis of acute and chronic diseases such as diabetes, cardiovascular diseases, pulmonary edema, and immune disorders [33]. On the other hand, the intensity and duration controlled hypoxia produces a beneficial effect through adaptation mechanism [34]. Many studies provide strong evidence that CIHH has a protective effect on heart, brain, and liver against ischemia/reperfusion or hypoxia/reoxygenation injury [2]. However, limited studies have been performed regarding the effect of CIHH on immune system function in immune diseases such as bronchial asthma, allergic dermatoses, and autoimmune thyroiditis [35]. Our present study demonstrated that CIHH treatment has a protective effect on experimental CIA in rats, manifesting as decrease in incidence of CIA, inhibition of synovial inflammation, and improvement of the pathological changes of synovium in CIA rats.

In this study, we investigated the effect of CIHH pre-treatment on CIA rat and got positive result, which suggests that CIHH might have a preventive action for RA in clinic. But we don’t know whether CIHH has treatment effect on RA. In other words, we do not know whether CIHH post-treatment has positive effect on CIA rats. It is the limitation of this study and need further investigation.

In summary, our studies demonstrated for the first time that CIHH pretreatment has a protective effect on CIA in rat through reducing the genesis of inflammatory cytokines TNF-а and IL-17, balancing the homeostasis of T-lymphocyte subsets, and modulating HIF-1α and NF-κB signal transduction pathways.
